# Aspects cliniques et étiologiques des intertrigos d’origine fongique à Abidjan (Côte d'Ivoire)

**DOI:** 10.11604/pamj.2019.33.198.19090

**Published:** 2019-07-12

**Authors:** Valerie Bedia-Tanoh Akoua, Pulchérie Christianne Marie Kiki-Barro, Abibatou Konaté, Etien Angora Kpongbo, Fulgence Kassi Kondo, Henriette Bosson-Vanga, Jean Sebastien Miezan Asouhoun, Djohan Vincent, William Yavo, Ignage Hervé Menan Eby

**Affiliations:** 1 Département de Parasitologie-Mycologie-Zoologie, Université Félix Houphouet-Boigny, Abidjan, Côte d'Ivoire; 2 Laboratoire de Parasitologie-Mycologie, Institut National de la Santé Publique, Abidjan, Côte d'Ivoire; 3 Laboratoire de Parasitologie-Mycologie, Centre de Diagnostic et de Recherche sur le Sida et les Autres Maladies Infectieuses, Abidjan, Côte d'Ivoire

**Keywords:** Intertrigos, étiologie fongique, Abidjan, Côte d´Ivoire, Intertrigos, fungal etiology, Abidjan, Ivory Coast

## Abstract

**Introduction:**

l'épidémiologie de ces affections surtout d'origine mycosique est peu connue en Côte d'Ivoire. Cette étude avait pour objectif de déterminer les différents aspects cliniques et fongiques des intertrigos.

**Méthodes:**

D'avril à octobre 2012, une étude transversale a été conduite dans le service de dermatologie clinique du Centre Hospitalier et Universitaire de Yopougon (Abidjan-Côte d'Ivoire). Elle a concerné les patients vus en consultation et présentant des lésions des plis, évocatrices d'une atteinte mycosique. Les prélèvements de sérosités par écouvillonnage ou de squames par raclage à la lame de Bistouri ont été réalisés au niveau des lésions. Les agents fongiques responsables de ces lésions ont été identifiés après culture du produit biologique.

**Résultats:**

Au total, 200 patients ont présenté des lésions évocatrices d'intertrigo d'origine fongique. L'âge moyen des patients était de 29,8 ans (écart type = 11,1 ans). Les intertrigos mycosiques ont représenté 6,7 % des motifs de consultation. Une prédominance féminine a été observée (76,7%). Les lésions siégeaient majoritairement au niveau de l'aine (40,8%) et des plis inter-fessiers (36,9%). Les symptomatologies les plus observées étaient la macération (52,4%) suivie de la brûlure (18,4%). Les intertrigos étaient causés dans 89,3% des cas par les levures, parmi lesquelles Candida albicans (33%) et Candida parapsilosis (19,4%) étaient prédominants.

**Conclusion:**

Les intertrigos d'origine mycosique affectent principalement les adultes jeunes de sexe féminin. Les lésions siègent préférentiellement au niveau des plis inguinaux et inter-fessiers. Les principaux agents étiologiques sont représentés par des levures du genre Candida.

## Abstract (en)

**Introduction:**

Epidemiology of these disorders, mainly caused by mycosis, is little known in the Ivory Coast. The aim of this study was to determine the different clinical aspects of intertrigos caused by fungal infections.

**Methods:**

We conducted a cross-sectional study in the Department of Clinical Dermatology at the University Hospital in Yopougon (Abidjan, Ivory Coast) from April to October 2012. The study involved the patients come to consultation with lesions in the folds suggesting a mycosis. Samples of serous fluid by swabbing or of scales by scrape cutting with the scalpel blade were performed at the level of the lesions. The fungal agents responsible for these lesions were identified after biological culture.

**Results:**

A total of 200 patients had lesions suggesting intertrigo caused by fungal infection. The average age of patients was 29.8 years (with a standard deviation of 11.1 years). Mycosis-related intertrigos accounted for 6.7% of reasons for consultation. A female predominance was observed (76.7%). Lesions mainly occurred in the groin area (40.8%) and in the intergluteal clefts (36.9%). The most observed symptoms were maceration (52.4%) followed by burning (18.4%). In 89.3% of cases, intertrigos were caused by yeasts, including Candida albicans (33%), and Candida parapsilosis(19.4%) which were predominant.

**Conclusion:**

Mycosis-related intertrigos mainly affect the young adults of female sex. Lesions mainly occur at the level of the inguinal folds and intergluteal clefts. The main etiological agents are yeasts (Candida).

## Introduction

L'augmentation de la prévalence des infections fongiques, lors des vingt dernières années, a profondément transformé l'attention portée à la mycologie médicale. Ces pathologies surviennent aussi bien chez des patients fragilisés (transplantation d'organes, greffes de moelle, chimiothérapies aplasiantes, immunosuppresseurs,) que chez les sujets immunocompétents et peuvent être profondes ou superficielles [1]. Les formes superficielles touchant les phanères, les muqueuses, la peau surtout au niveau des plis sont appelées intertrigos. L'intertrigo se définit comme une atteinte inflammatoire de la peau au niveau des plis cutanés principalement reconnue par un érythème plus ou moins intense pouvant aboutir à de graves complications engageant même le pronostic vital du patient [2, 3]. Les étiologies des intertrigos sont multiples et variées aussi bien chez l'adulte que chez l'enfant. Les conditions particulières, telles que le frottement entre les surfaces des plis et la macération due à l'augmentation de la température locale, sont des facteurs favorisants l'inflammation et la surinfection. Le défaut ou l'excès d'hygiène, les vêtements serrés, l'obésité, le diabète et certains traitements mal adaptés sont aussi des facteurs de déclenchement et d'entretien des lésions [4]. Le diagnostic étiologique repose sur une démarche diagnostique cohérente qui permettra par la suite une prise en charge thérapeutique spécifique du patient. La fréquence des champignons agents des intertrigos varie en fonction des différences régionales, des habitudes culturelles, des flux migratoires et aussi change dans le temps [3]. À ce jour il existe peu d'études documentées sur les intertrigos d'origine fongique en Côte d'Ivoire. L'objectif du présent travail est d'établir le profil clinique et fongique de ces affections dans un Service de Dermatologie Clinique à Abidjan.

## Méthodes

**Type, lieu et population d'étude**: il s'est agi d'une étude transversale, conduite chez des patients sans distinction d'âge et de sexe vus en consultation dans le service de dermatologie clinique du Centre Hospitalier et Universitaire de Yopougon (Abidjan-Côte d'Ivoire) pendant une durée de 7 mois (avril à octobre 2012). Les examens mycologiques ont été réalisées au Centre de Diagnostic et de Recherche sur le SIDA et les autres maladies infectieuses (CeDReS) sis au sein du CHU de Treichville.

**Méthodes**: pour mener à bien cette étude, le consentement éclairé écrit des patients et l'accord du chef de Service de Dermatologie Clinique du CHU de Yopougon ont été obtenus. Les données biologiques et cliniques ont fait l'objet d'une stricte confidentialité. Les patients consentant, venus pour une consultation au service de dermatologie du CHU de Yopougon sans distinction d'âge ou de sexe ont été soumis à un examen clinique complet. Ceux présentant des lésions des plis, évocatrices d'une atteinte mycosique ont fait l'objet d'un prélèvement et d'un interrogatoire. Le questionnaire a permis d'obtenir les données socio-démographiques, les données sur l'activité sportive, l'hygiène quotidienne, le port de vêtements serrés et de chaussures fermés, l'atteinte familiale et la présence d'animaux domestiques. Sur les lésions cutanées sèches, des squames ont été prélevés à l'aide d'une lame de Bistouri et mises dans une boite de Pétri stérile. En ce qui concerne les lésions suintantes, le prélèvement a été effectué à l'aide de deux écouvillons stériles. Ensuite, un examen direct microscopique (à l'objectif x10 puis à l'objectif x40) du produit pathologique a été effectué après éclaircissement à la potasse à 30% (pour les squames) et dans du sérum physiologique (pour les écouvillons). La culture a été obtenue après ensemencement du produit pathologique sur deux milieux de Sabouraud: l'un additionné de Chloramphénicol (SC) et l'autre additionné de Chloramphénicol et d'Actidione (SAC), qui ont été incubés à 27°C pendant une à trois semaines. Toutes les cultures positives, ont fait l'objet d'identification.

**Identification des agents fongiques**: l'identification des levures s'est faite après 24 à 48 heures. L'examen macroscopique des colonies a permis d'observer des colonies crémeuses, luisantes ou mates, de couleur blanchâtre. Une étape de confirmation a été effectuée par un examen direct dans du sérum physiologique. L'identification des espèces de levure a été réalisée selon la démarche classique: d'abord le test de blastèse, ensuite le test de chlamydosporulation sur milieu PCB (Bio-rad^®^), puis l'auxanogramme a été réalisé en utilisant le système d'identification des levures API 20 C AUX (Bio Mérieux ®). Quant aux dermatophytes, ils ont été identifiés sur la base des caractères culturaux (macroscopie et microscopie des cultures).

**Analyse statistique**: les données ont été saisies sur le logiciel Excel, codées et transférées dans le logiciel Statistical Package for Social Science version 16.0. (SPSS) pour analyses statistiques. Pour la comparaison des proportions, nous avons utilisé le test de Khi-deux ou de Fisher au seuil α = 5%.

## Résultats

**Caractéristiques socio-démographiques**: pendant la période de l'étude, 1517 patients ont été vus en consultation dans le service de dermatologie clinique. Le sexe féminin était prédominant (sex-ratio = 0,3). L'âge moyen des patients était de 29,7 ans (écart-type = 11,1 ans) avec des extrêmes allant de 1 mois à 58 ans. Deux tiers des patients (60,5 %) avaient un âge compris entre 15 et 30 ans (Tableau 1).

**Tableau 1:** Caractéristiques socio-démographiques de la population d’étude

Variables	Nombre (N)	Pourcentage (%)
**Age (ans)**		
< 15	9	4,5
15 – 30	121	60,5
30-45	46	23
> 45	24	12
**Sexe**		
Masculin	65	32,5
Féminin	135	67,5

**Figure 1:**
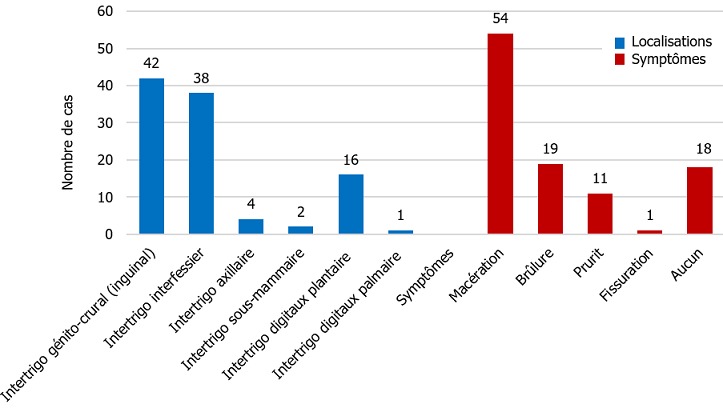
Aspect clinique de l’intertrigo

**Localisation des intertrigos et aspects cliniques**: la majorité des patients (83,5 %), avait des lésions au niveau des grands plis, notamment génito-cruraux (40,8 %), inter fessiers (36,9%) et axillaires (3,9 %). L'intertrigo inter orteil a représenté 15,5% des atteintes. Au plan clinique, la macération était présente dans 52,4% des cas. D'autres symptômes tels que les brûlures (18,4 %), le prurit (10,7 %), ont été les plus observés (Figure 1).

**Prévalence de l'intertrigo**: au total, 200 patients sur 1517 présentaient des lésions des plis évocatrices d'un intertrigo d'origine fongique. Soit une prévalence clinique de 13,2%. Au plan mycologique, la culture a été positive dans 51,5% des cas (103/200) soit une prévalence globale de 6,7%. Le sexe féminin était le plus touché (76,7%), surtout les femmes âgées de 15 à 30 ans (53%) avec une différence statistiquement significative (p=0,024) (**Tableau 2**). Les élèves et étudiants étaient plus représentés (34,9%).

**Tableau 2:** Répartition des cas d’intertrigos fongique selon les caractéristiques socio-démographiques

Variables	Nombre (N)	Pourcentage (%)	p-value
**Age (ans)**			
< 15	5	4,8	0,107
15 - 30	70	68	
30 – 45	20	19,4	
> 45	8	7,8	
**Sexe**			
Masculin	24	23,3	0,024
Féminin	79	76,7	
**Catégories socio-professionnelles**			
Elèves / Etudiants	36	34,9	
Secteur informel	23	22,3	0,229
Sans emploi	20	19,4	
Fonctionnaires	15	14,5	
Ménagères	6	5,8	
Agriculteurs	3	0,9	

**Figure 2:**
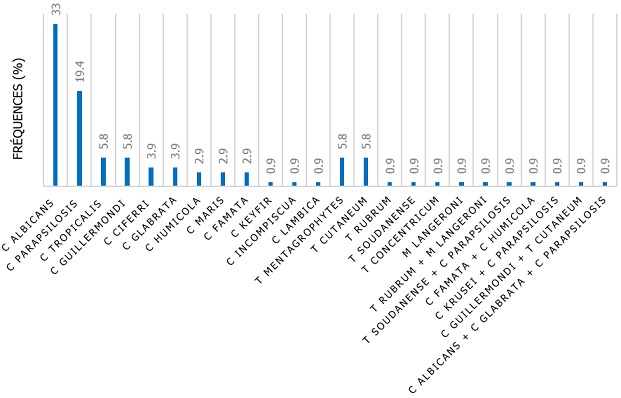
Étiologie fongique des cas d’intertrigos

**Espèces fongiques identifiées**: le diagnostic mycologique a permis d'objectiver 89,3% et 10,7% des intertrigos respectivement d'origine candidosique et dermatophytique. Six cas d'associations ont été notés (Figure 2). À l'exception des plis axillaires, *Candida albicans* était majoritairement retrouvé au niveau des plis interdigitaux (50 %), des plis inguinaux et interfessiers (38,5%), et des plis inter-orteils (21,5 %) (Tableau 3). *C. parapsilosis* qui n'épargnait aucun pli, occupait le second rang après *C. albicans*. Quant à *C. tropicalis*, il a été retrouvé principalement au niveau de plis axillaires (16,6%). À côté de ces principales espèces, neuf autres espèces du genre *Candida* ont été isolées des plis inguinaux et inter fessiers (Tableau 3). En dehors des levures du genre *Candida*, des dermatophytes (Tableau 3), ont été principalement isolées au niveau des plis inter-orteils. Il s'agit principalement de *T. mentagrophytes* (groupe *mentagrophytes*) (26,3 %). *M. audouini i*variété *langeronii, T. soudanens*e et *T. concentricum* ont été retrouvés à la même fréquence (5,2 %). En plus des plis inter-orteils, c'est au niveau des plis inguinaux et interfessiers qu'a été isolé le plus de dermatophytes, notamment *T. concentricum* (4,8 %). Un cas de *T. rubrum* a été retrouvé au niveau des plis axillaires.

**Tableau 3:** Étiologies fongiques de l’intertrigo et siège de la lésion

Espèces candidosiques	Fréquences selon le siège (%)			
	Plis inguinaux et interfessiers	Plis axillaires	Plis interorteils	Plis interdigitaux
*C albicans*	38,5	-	21,5	50
*C parapsilosis*	19,3	16,6	10,5	50
*C tropicalis*	7,2	16,6	5,2	-
*C guillermondi*	6	16,6	-	-
*C ciferri*	3,6	-	-	-
*C glabrata*	4,8	-	-	-
*C humicola*	3,6	-	-	-
*C maris*	2,4	16,6	-	-
*C famata*	3,6	-	5,2	-
*C keyfir*	1,2	-	-	-
*C incompiscua*	-	-	5,2	-
*C lambica*	1,2	-	5,2	-
**Espèces dermatophytiques**				
*M. langeroni*	1,2	-	5,2	-
*T. rubrum*	-	1,6	-	-
*T. mentagrophytes*	1,2	-	26,3	-
*T. soudanense*	-	-	5,2	-
*T concentricum*	4,8	-	5,2	-

## Discussion

Le diagnostic clinique de l'intertrigo d'origine fongique est généralement peu aisé. Sur la seule base de l'aspect clinique, la mycose peut être diagnostiquée à tort du fait de la diversité étiologique de l'intertrigo [3, 4]. Ainsi, dans notre étude, le diagnostic clinique a été couplé au diagnostic mycologique notamment la culture permettant un diagnostic précis de la maladie. Ainsi, une prévalence de 6,7% a été obtenue. En Afrique, comme ailleurs dans le monde, l'intertrigo des grands plis le plus courant, est celui des plis inguinaux [3, 5]. Dans notre série, le taux de prévalence des intertrigos relativement élevé pourrait être dû, au port régulier de sous-vêtements serrés entrainant ainsi une macération. En revanche, nos données contrastent avec celles obtenues au Japon et en Australie où l'intertrigo inguinal semble plus fréquent chez le sujet de sexe masculin (86 hommes contre 26 femmes) [6, 7]. La tendance à une fréquence moindre de l'atteinte des plis axillaires (3,9%), sous-mammaires (1,9%) et inter digitaux palmaires (0,9%) a également été observée dans une étude conduite en milieu hospitalier à Dakar [3]. Dans cette étude, les auteurs notent des fréquences de 20,4%, 12%, 6% et 7,8% respectivement de lésions des plis axillaires, sous-mammaires et inter-fessiers.

L'atteinte préférentielle des plis interdigitaux plantaires est fréquemment rapportée. Ainsi, en Côte d'Ivoire, Barro *et al.* ont rapporté une prévalence de 76,9 % chez les élèves gendarmes, et deux études menées au Sénégal [3-8], mentionnaient des taux plus élevés que les nôtres, respectivement 65,3% et 25,5%. En revanche, un taux de prévalence plus faible au nôtre (5,5%) est noté dans une prison de Ouagadougou au Burkina Faso [5]. Cet éclectisme des agents fongiques pour les plis inter-orteils serait en rapport avec l'humidité constante qui y règne, favorisant ainsi la survenue de l'intertrigo [9, 10]. Au plan étiologique, contrairement à de nombreuses études portant sur les dermatomycoses qui notaient une fréquence élevée des intertrigos d'origine dermatophytique [3, 5, 8], la présente étude a rapporté une prédominance des intertrigos candidosiques. La prépondérance de *Candida albicans* au niveau des plis génitaux n'est pas retrouvée en milieu hospitalier sénégalais [3]. Cependant la forte implication de *C. albicans* dans la pathologie fongique des intertrigos n'est pas un fait nouveau. Notre résultat pourrait être le fait d'une colonisation secondaire de *C. albican*s à partir des muqueuses génitales. En effet, vivant à l'état commensal dans les muqueuses génitales, *C. albicans* peut passer à l'état pathogène sous l'influence de divers facteurs favorisants.

Les espèces de dermatophytes retrouvées dans notre série étaient dominées par les espèces anthropophiles. En Europe, chez des patients de sexe masculin originaires d'Autriche l'étiologie fongique de l'intertrigo des parties génitales étaient exclusivement dermatophytique et dominée par des espèces zoophiles. En effet, dans de nombreuses études, les espèces zoophiles étaient fréquentes, dominées par *Microsporum canis* et *Trichophyton interdigitale* [11, 12]. Les espèces anthropophiles moins fréquentes, étaient représentées par *T. rubrum* et *T. tonsurans*. Le climat humide et tropical, ainsi que le mode de vie des sujets de notre étude (absence de contact avec les animaux, le type d'activité physique) pourraient expliquer cette différence. L'espèce *Trichophyton rubrum* semble être la plus retrouvée dans de nombreuses études réalisées dans diverses zones géographiques du globe [13-18]. Des cas d'infections mixtes ont été observés (5,4%). Il peut donc se poser un problème de prise en charge thérapeutique. En effet, le traitement des intertrigos est différent en fonction du germe responsable de l'infection. En outre, le choix des antifongiques doit être bien adapté et tenir compte des résistances éventuelles, la localisation du foyer infectieux et la nature du terrain sur lequel survient l'infection. Il dépend également de la pharmacocinétique de l'antifongique, de ses effets secondaires. Par exemple, la nystatine est efficace seulement pour l´intertrigo candidal tandis que le clotrimazole, kétoconazole, oxiconazole ou éconazole peuvent être utilisés pour les infections à Candida et à dermatophyte [19].

## Conclusion

Les intertrigos d'origine mycosique touchent principalement les adultes jeunes de sexe féminin. Les lésions siègent préférentiellement au niveau des plis inguinaux et inter-orteils. Les principaux agents étiologiques sont représentés par des levures du genre *Candida*.

### État des connaissances actuelles sur le sujet

- Peu de données sur l'intertrigo mycosique en Côte d'Ivoire;
- Étiologie et facteurs de risques ou favorisants des intertrigos;
- Étiologies fongiques et facteurs favorisant les intertrigos inter-orteils chez les gendarmes à Abidjan (Côte d'Ivoire).

### Contribution de notre étude à la connaissance

- Obtenir des données en établissant le profil clinique et fongique des intertrigos mycosiques à Abidjan;
- Actualisation de l'étiologie fongique des intertrigos;
- Détermination de la localisation des intertrigos d'origine fongique.

## Conflits d’intérêts

Les auteurs ne déclarent aucun conflit d’intérêts.
